# Langerhans cells as targets of rabies virus immune evasion

**DOI:** 10.1099/jgv.0.002318

**Published:** 2026-09-17

**Authors:** Keshia Kroh, Leanne C. Helgers, Teunis B. H. Geijtenbeek, Carmen W. E. Embregts, Corine H. GeurtsvanKessel

**Affiliations:** 1 Department of Viroscience, Erasmus Medical Centre, Rotterdam, The Netherlands; 2 Department of Experimental Immunology, Amsterdam UMC, University of Amsterdam, Amsterdam, The Netherlands; 3 Amsterdam Institute for Infection Diseases and Immunology, Amsterdam UMC, University of Amsterdam, Amsterdam, The Netherlands

**Keywords:** antiviral immunity, Langerhans cells (LCs), rabies virus (RABV), skin immunity

## Abstract

Rabies virus (RABV) is a neurotropic virus of the genus *Lyssavirus* that causes a nearly always fatal encephalitis in humans once it reaches the central nervous system. Dog bites are a major transmission route of RABV, and following superficial skin exposure, RABV first encounters epidermal Langerhans cells (LCs), yet their role in early infection remains poorly understood. Here, we show that exposure of immature human epidermal LCs to RABV leads to RABV association with cells in the absence of productive viral replication. RABV-exposed LCs display reduced cytokine and type I IFN responses and lack induction of IFN-stimulated genes, indicating suppression of antiviral signalling pathways. Together, these findings suggest that RABV is efficiently taken up by immature LCs, which dampens the antiviral immune responses of LCs, potentially facilitating immune evasion at the skin entry site.

## Introduction

Infection with rabies virus (RABV), a neurotropic virus of the genus *Lyssavirus* within the family *Rhabdoviridae* and order *Mononegavirales* [1], causes fatal encephalitis in humans once it reaches the central nervous system (CNS). Rabies remains a significant global public health problem, causing an estimated 59,000 human deaths annually worldwide, with the highest burden in Asia and sub-Saharan Africa [2–4]. While rabies can be effectively prevented through rapid post-exposure prophylaxis (PEP), access to vaccines and rabies immunoglobulins remains limited in many endemic regions, resulting in substantial preventable mortality. Rabies is fatal once clinical symptoms develop, disproportionately affecting children and populations with limited access to PEP [2–4]. The majority of human rabies cases (>99%) result from dog bites and transmission typically occurs through introduction of infectious saliva via the bite, causing viral exposure of different skin layers, including the epidermis and dermis, and the muscle [5]. However, rabies can also be transmitted through superficial bites or scratches from dogs or other animals, which can result in viral exposure of only the superficial skin layers [6, 7]. Moreover, recent studies have shown that RABV is able to reach neurons of the CNS even when the keratinocyte barrier remains intact [8], and clinical rabies cases in the absence of a clear scratch or bite have been documented. These observations implicate the skin as important for RABV entry [9, 10], as nerve endings in the skin are thought to serve as the primary route of RABV entry into the CNS [11].

The skin is an immunologically active barrier populated by diverse resident immune cells, including macrophages, dermal dendritic cells and epidermal Langerhans cells (LCs) [12–16]. Epidermal LCs are antigen-presenting cells and are the first immune cells to encounter invading pathogens that breach the skin surface. Upon activation, LCs migrate to draining lymph nodes to initiate adaptive immune responses, thereby playing a critical role in early antiviral defence [12]. IFN responses, particularly the induction of IFN-stimulated genes (ISGs), are central to antiviral immunity, restricting viral replication and shaping both innate and adaptive immune responses [17]. Despite these defences, RABV can reach the CNS without triggering effective immune activation, suggesting that the virus interferes with early skin immune responses [18, 19].

RABV uses multiple mechanisms to evade and suppress the immune system [20–22]. Recent studies have further shown that RABV can suppress macrophage activation without establishing productive infection and fails to induce sufficient dendritic cell (DC) activation, highlighting active viral modulation of myeloid immune responses [23–26]. Given their strategic localization in the epidermis and their critical role in initiating immune responses, LCs are likely among the first immune cells to encounter RABV following superficial skin exposure. However, the contribution of LCs to early RABV sensing remains poorly understood. A better understanding of early RABV interactions with skin immune cells, particularly LCs, is essential for elucidating mechanisms of immune evasion at the site of entry, which remains poorly characterized, especially in cases of superficial exposures. In this study, we have characterized the effects of RABV exposure on human immature LCs by determining RABV infection and the corresponding cytokine response.

Immature LCs exposed to RABV showed an increase in RABV-positive cells, as observed by flow cytometry, while viral titres remained unchanged during the course of exposure. Our data indicate that prolonged exposure to RABV does not result in productive viral replication but likely leads to an abortive infection in immature LCs. Instead, exposure to RABV results in reduced cytokine and type I IFN production and a lack of strong ISG induction. These findings suggest that RABV is capable of suppressing antiviral mechanisms in immature LCs as well.

## Methods

### Virus production and quantification

SHBRV and dogRV are pathogenic street RABV strains and were isolated from fatal human cases related to contact with a bat and a dog, respectively [27, 28]. The attenuated SAD P5 strain was obtained from Institute Pasteur, Paris, France, through the European Virus Archive Global repository (SAD P5/88 Potsdam, isolate 9509TCH, Ref-SKU 014 V-01931). Viruses were cultured on SH-SY5Y cells as described previously [26] and were used at passage numbers two to five. Infectious virus particles were quantified using the endpoint dilution method of Reed and Muench [29] using SH-SY5Y cells, as described previously [26]. The TCID_50_ of virus stocks were 1.11E+07 (dogRV), 1.11E+08 (SAD P5) and 7.31E+06 (SHBRV). Corresponding cycle threshold values of viral RNA were measured by reverse transcription quantitative real-time polymerase chain reaction (RT-qPCR) as described previously [8] as 19.0 (dogRV), 18.0 (SAD P5) and 19.3 (SHBRV) (primers F: 5′-ATGTAACACCYCTACAATG-3′; R: 5′-GCMGGGTAYTTGTACTCATA-3′). Viral titres in LC supernatants were expressed as Δlog_10_TCID_50_ ml^−1^, representing the log_10_-fold change at each time point relative to the titre measured at 1 h post-infection (hpi), following removal of the inoculum.

### Immature LC isolation

Human skin tissues were obtained from healthy donors undergoing corrective breast or abdominal surgery. Isolated immature LCs were purified as described [13, 30]. Briefly, to obtain epithelial sheets, skin tissue was treated over night with dispase II (1 U ml^−1^, Roche Diagnostics), after which the epidermis was separated from the rest of the tissue. Next, skin epithelial sheets were incubated in PBS containing DNase I (20 units ml^−1^, Roche Applied Science) and trypsin (0.05%, Becton Dickinson) for 30 min at 37 °C to obtain a single-cell suspension. Trypsin digestion was inactivated with FCS. Single cells were washed, layered on a Ficoll (Axis‐shield) gradient, and LCs were purified using cluster of differentiation (CD)1a‐labelled immunomagnetic microbeads according to the manufacturer’s instructions (Miltenyi Biotec). Cells were frozen in Iscove's Modified Dulbecco's Medium (IMDM) (Thermo Fisher Scientific) containing 10% DMSO and 10% FCS (Invitrogen) and stored in liquid nitrogen.

### Infection

LCs were placed in 96-well U-bottom plates (Greiner Bio-One) at a density of 1×10^5^ cells per well in LC culture medium [IMDM (Capricorn Scientific), 10% (v/v) FCS (Sigma-Aldrich), 2 mM l-glutamine (Capricorn Scientific), 1× Penicillin/Streptomycin (Capricorn Scientific) and 20 µg ml^−1^ gentamycin (Centrafarm)]. For infection, medium was removed, and cells were resuspended in the respective virus diluted in serum-free medium. A m.o.i. of 1 and 10 were used, based on TCID_50_. LCs were incubated with conditioned medium harvested from uninfected SH-SY5Y cells to control for a potential effect of cell-derived soluble factors present in viral stocks. No significant differences in cytokine secretion were observed. After 1 h of incubation at 37 °C and 5% CO_2_, the inoculum was removed, and cells were carefully washed once with culture medium and cultured for 48 h in culture medium. Supernatants for virus titrations were collected after washing and at 24 and 48 hpi.

### Flow cytometry staining

At 48 hpi, cells were collected and stained for flow cytometry as described previously [26]. Zombie Violet Fixable Viability Stain (BioLegend) was used for live/dead staining. LCs were gated based on viability and staining for CD1a (BV510 mouse anti-human CD1a, clone HI149, BD Biosciences) and CD207/Langerin (PE-Cyanine7 anti-mouse/human CD207, clone 4C7, BioLegend). Infection percentages were assessed based on staining for intracellular RABV nucleoprotein (RABV-N; FITC conjugate, Fujirebio). Cells were fixed using the Cytofix/Cytoperm kit (BD Biosciences) and analysed with a BD FACSLyric flow cytometer (BD Biosciences) and FlowJo V10 software (FlowJo LLC).

### Cytokine quantification

Culture supernatants were harvested at 48 hpi and stored at −70 °C. Cytokine concentrations were quantified using the LEGENDplex Human Anti-Virus Response Panel 13-plex bead-based immunoassay kit (BioLegend) according to the manufacturer’s instructions. Beads were fixed and washed prior to measurement using the Cytofix/Cytoperm kit (BD Biosciences), as described previously [26]. Fluorescence signals were analysed with a BD FACSLyric flow cytometer (BD Biosciences) and the LEGENDplex Data Analysis Software Suite (BioLegend). Values outside the detection limits were extrapolated based on standard curves and zero values were replaced with half the assay detection limit. Cytokine concentrations were log-transformed, and donor-wise log_₂_ fold-changes relative to matched mock controls were calculated. Donor-wise log_₂_ fold-changes relative to mock were tested against zero using one-sample t-tests, and *P*-values were adjusted using the Benjamini–Hochberg false discovery rate.

### Statistical analysis

Pairwise t-tests for repeated-measures data were performed to test for statistically significant differences in percentages of RABV-N-positive cells. Δlog_10_TCID_50_ ml^−1^ values at 24 and 48 hpi were tested against zero (no difference) using one-sample t-tests for each strain and m.o.i. individually. For cytokine data, donor-wise log_₂_ fold-changes relative to mock were tested against zero using one-sample t-tests, and *P*-values were adjusted using the Benjamini–Hochberg false discovery rate. Adjusted *P*-values of <0.05 were considered statistically significant.

### Data visualization

Data were processed and visualized using the RStudio environment, version 2025.05.1 (Posit PBC) and packages tidyverse [31] and ggplot2 [32].

## Results

### Limited RABV replication in LCs despite intracellular viral protein expression

**Fig. 1.**
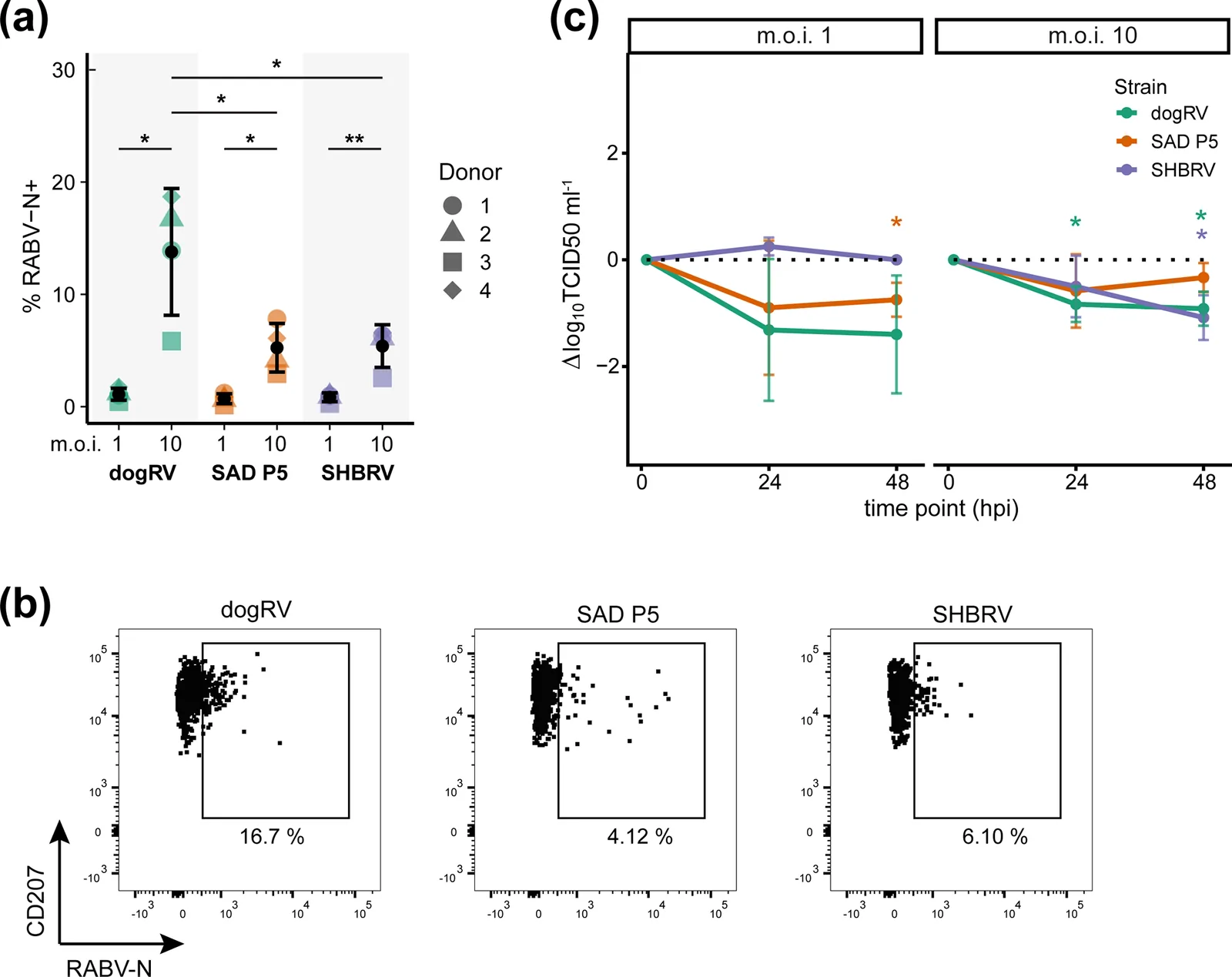
Susceptibility of LCs to RABV infection. (a) LCs were exposed to different RABV strains and infection was determined by intracellular RABV-N staining. Percentages of live LCs that were positive for intracellular RABV-N staining at 48 hpi, measured by flow cytometry. Statistical significance was determined by pairwise t-tests, and significance values are shown for comparisons within the same strain or the same m.o.i. (**b)** Representative dot plots showing the RABV-N-positive cell fractions of LCs exposed to the three different RABV strains at an m.o.i. of 10. (**c)** Viral titres in LC supernatants, collected after washing (1 hpi), at 24 and at 48 hpi. Expressed as Δlog_10_TCID_50_ ml^−1^ relative to the residual titre measured at 1 hpi. Lines show mean values of *n* = 4 donors ± sd. One-sample t-tests were performed to determine if Δlog_10_TCID_50_ ml^−1^ at 24 and 48 hpi differed significantly from 0 for each strain and m.o.i. separately. *P<0.05, **P<0.01.

LCs were exposed to street RABV strains SHBRV and dogRV and the vaccine strain SAD P5 at m.o.i.s of 1 and 10, washed and cultured for 48 h. Flow cytometry analysis showed that at an m.o.i. of 1, the average percentages of cells positive for intracellular RABV-N were 1.1% (dogRV), 0.8% (SHBRV) and 0.7% (SAD P5) (Fig. 1a). Moreover, these percentages increased to averages of 13.8% (dogRV), 5.4% (SHBRV) and 5.2% (SAD P5) when exposed to an m.o.i. of 10 (Fig. 1a). Internalization of the virus was visualized by means of confocal microscopy (Fig. S1, available in the online Supplementary Material). The RABV-N fluorescence intensity was comparably dim for LCs exposed to SHBRV and dogRV, whereas a higher RABV-N fluorescence intensity was detected in a fraction of LCs exposed to SAD P5 (Fig. 1b). Despite the detection of intracellular RABV-N, no increase in secreted infectious virus particles was detected (Fig. 1c). Viral titres in culture supernatants of virus-exposed LCs were determined based on the infectivity of the highly susceptible neuronal cell line SH-SY5Y and were not increased above baseline at 48 hpi.

### RABV exposure leads to decreased cytokine secretion by LCs

**Fig. 2.**
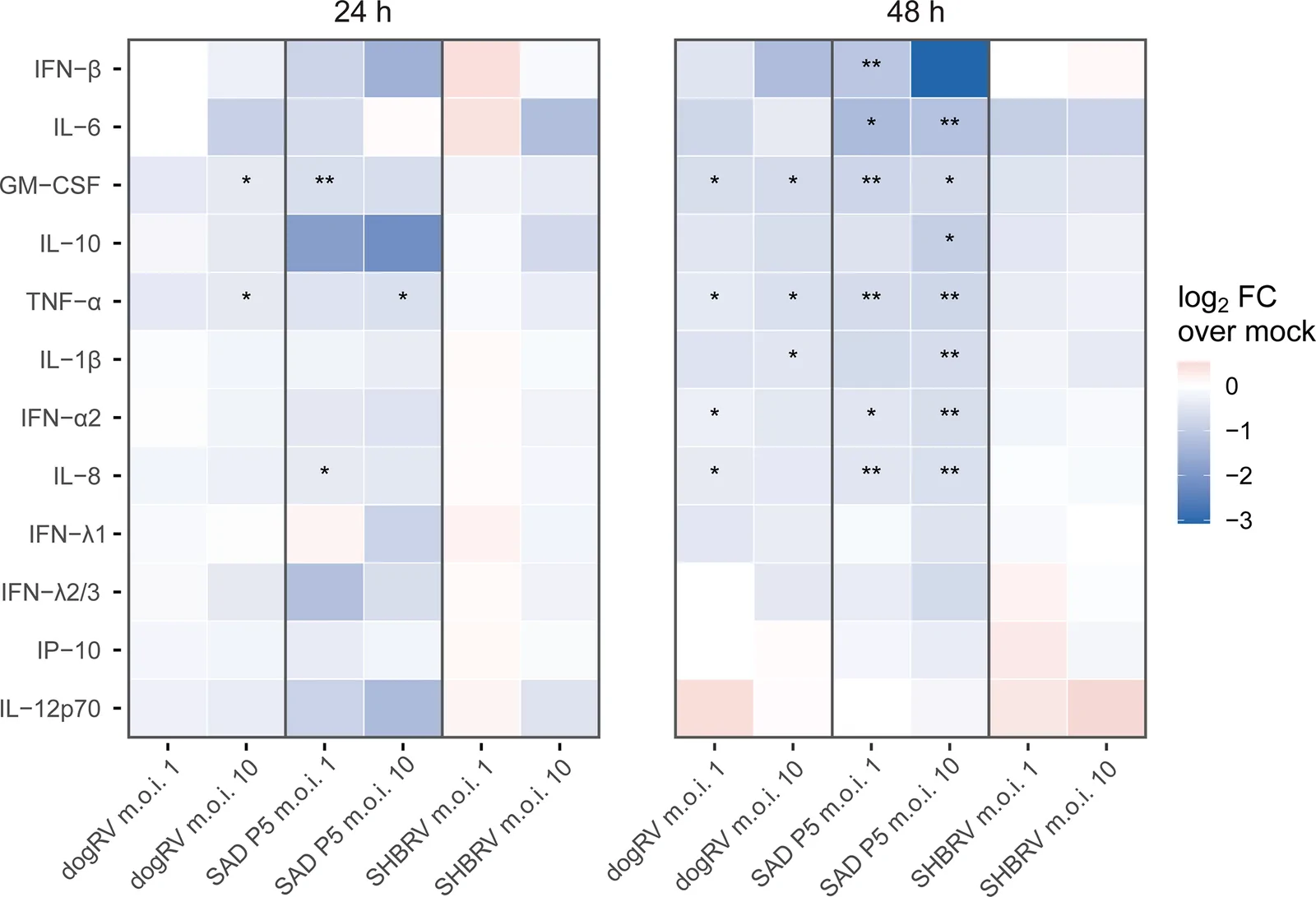
Cytokine responses of LCs following exposure to RABV. Immature LCs were exposed to the indicated RABV strains at an m.o.i. of 1 or 10, and supernatants were collected at 24 and 48 hpi. Cytokine concentrations were quantified using a multiplex bead-based assay and are shown as mean log_2_ fold-changes (log_2_ FC) relative to donor-matched mock controls. Statistical significance was determined using one-sample t-tests against zero, with Benjamini–Hochberg correction for multiple testing. *n =* 4 individual donors. *P_adj_<0.05, **P_adj_<0.01.

To investigate whether RABV has an immunomodulatory effect on LCs, cytokine expression was determined in culture supernatants at m.o.i.s of 1 and 10 at 24 and 48 hpi (Fig. 2). Overall, both at 24 and 48 h, the concentrations of most cytokines and IFN responses were decreased in the supernatants of dogRV- and SAD P5-exposed LCs compared to the mock controls. We did not observe any significant changes in immune responses following SHBRV exposure. Mean log_2_ fold-changes were generally more pronounced at 48 hpi than at 24 hpi and, at 48 hpi, ranged from −1.27 (IL-6, SAD P5 m.o.i. 1) to 0.53 (IL-12p70, SHBRV m.o.i. 10); with the exception of IFN-β, which showed a pronounced average 3.07-fold decrease, following exposure to SAD P5 at an m.o.i. of 10. DogRV exposure led to significantly decreased concentrations of cytokines granulocyte-macrophage colony-stimulating factor (GM-CSF), TNF-α, IFN-α2 and IL-8 at an m.o.i. of 1 compared to the mock. The concentrations of GM-CSF, TNF-α and IL-1β were significantly decreased at an m.o.i. of 10. SAD P5 exposure led to significantly decreased concentrations of IL-1β, TNF-α, IL-8, IFN-α2, IFN-λ2/3, GM-CSF, IFN-β and IL-10 at an m.o.i. of 10 and of IL-1β, TNF-α, IL-8, IFN-α2, GM-CSF and IFN-β at an m.o.i. of 1. Interestingly, we also observed the absence of upregulation of the ISG interferon gamma-induced protein 10 (IP-10). Modulation of cytokine concentrations following SHBRV exposure was minor and not statistically significant. Raw cytokine measurements and individual data points are provided in Fig. S2.

## Discussion

In this study, we investigated the susceptibility of skin-resident immature LCs to RABV infection and showed that immature LCs are able to take up RABV. However, viral titres remained unchanged during the course of exposure, indicating a non-productive RABV infection of immature LCs. Moreover, we observed that the cytokine production of virus-exposed LCs was decreased compared to mock-exposed LCs, hinting towards suppression of the induced immune response.

Unchanged viral titres in RABV-exposed LC cultures are in contrast with recent observations showing that exposure of dendritic cells and keratinocytes resulted in similar proportions of ~10% RABV-N-positive cells accompanied by a 1–2 log increase in viral titres [8, 26]. The increase in viral titres indicates a robust productive RABV infection of these cell types. Despite comparable percentages of RABV-N-positive cells, LCs failed to support productive RABV replication, suggesting the presence of intrinsic restriction mechanisms that limit post-entry steps of the viral life cycle. This mechanism has been well studied in the context of human immunodeficiency virus-1, where the viral replication cycle is restricted due to internalization of the virus in Birbeck granules via the surface receptor langerin [13–15]. Our findings suggest the possibility that LCs may utilize overlapping restriction strategies to limit RABV replication. However, further studies into the involvement of langerin and other restriction mechanisms in limiting RABV replication within immature LCs are necessary.

Interestingly, while RABV replication seems to be restricted in LCs, viral exposure nonetheless suppressed the LC immune responses by reducing cytokine production and type I IFN responses. In addition, we observed a lack of ISG induction (IP-10). These observations suggest that RABV is able to evade early antiviral immune responses without requiring productive infection. Such immune modulation aligns with previous observations in macrophages, where RABV suppresses immune activation independently of replication, and supports a broader strategy by which RABV targets myeloid cells to dampen innate immune defences at the site of entry [24]. Together, these findings suggest that LCs act as a restrictive but immunologically vulnerable cell population during early RABV exposure, possibly capable of limiting viral replication yet susceptible to RABV-induced immune suppression. Elucidating these mechanisms may provide important insights into immune evasion by RABV at the location of entry during early infection.

In a previous study, RABV infection of keratinocytes triggered an activating response, as evidenced by antiviral cytokine and IFN secretion and an increased surface expression of intercellular adhesion molecule (ICAM)-1 and HLA-ABC [8]. Because LCs interact closely with keratinocytes and rely on keratinocyte-derived cytokines for adequate activation, crosstalk between these cell types likely shapes early immune responses in the epidermis [33, 34]. For instance, in an experimental dengue virus infection using human skin explants, the pro-inflammatory response of keratinocytes promoted the recruitment and infection of LCs [35]. Future work should take into account the bidirectional communication between keratinocytes and LCs to determine whether RABV is able to successfully evade immune responses initiated in the skin. In addition, the fate of the RABV-negative population remains unclear. These cells may have failed to internalize RABV antigens or rapidly degraded viral material below the detection limit. However, this cannot be determined from the present study and represents an important distinction for future mechanistic investigations.

To assess whether the effect of RABV on LCs is strain-dependent, we included three RABV strains of different origins in this study. The observation that only dogRV and SAD P5 suppress LC cytokine and type I IFN response, whereas SHBRV does not, suggests that immune suppression is a partially conserved mechanism of different RABV strains. This strain-specific adaptation may facilitate viral progression from the skin to the CNS and could potentially explain differences in disease progression among RABV isolates. Notably, suppression was most pronounced for the SAD P5 strain. This strain is used as a live virus oral vaccine for foxes and, as such, does not cause disease but instead induces a protective antibody response [36]. The strain has been developed through serial passaging in cell culture and animals, but little is known about the mechanism by which it triggers immune responses [37]. It is possible that this strain exhibits its immunostimulatory effect on other cell types or at later stages of disease. The pathogenic dogRV strain also significantly reduced antiviral cytokine secretion in LCs, which is in line with the limited induction of pathogen defence responses in keratinocytes and dendritic cells that was observed in previous studies [8, 26]. Combined, these observations suggest that dogRV has higher immune evasion capacity compared to the other pathogenic strain SHBRV.

Beyond human disease implications, understanding LC interactions with RABV has broader relevance for transmission dynamics. Dogs, the principal reservoir, transmit RABV efficiently through bites and scratches. If RABV similarly suppresses LC antiviral responses in canine skin, this could facilitate viral progression from the bite site to sensory neurons and subsequent salivary gland, resulting in enhanced transmission efficiency. Alternatively, species-specific differences in LC responses to RABV could explain differential reservoir competence across animal hosts.

In conclusion, our results indicate that immature LCs might restrict RABV replication while remaining vulnerable to viral suppression of antiviral and IFN responses. This might prevent LCs from initiating effective adaptive immunity at the site of RABV entry, contributing to the characteristic ‘silent’ progression of rabies during the initial stages of infection [38]. However, the underlying mechanism remains unresolved, and future studies employing receptor blocking and maturation-stage comparisons would help clarify whether restriction results from differential receptor expression, rapid innate signalling or other factors. In addition, future studies should investigate how LC infection and suppression influence key functions, such as viral dissemination, antigen presentation, migration to lymph nodes and T-cell priming. The immune response in the skin is poorly characterized despite its central role as virus entry site, so these insights are essential for future PEP development and for understanding the often overlooked skin route of RABV infection.

## Supplementary material

10.1099/jgv.0.002318 Fig. S1.

## Abbreviations

CD: cluster of differentiation
CNS: central nervous system
DC: dendritic cell
GM-CSF: granulocyte-macrophage colony-stimulating factor
hpi: h post-infection
IP-10: interferon gamma-induced protein 10
ISG: IFN-stimulated gene
LC: Langerhans cell
PEP: post-exposure prophylaxis
PEP: post-exposure prophylaxis
RABV: rabies virus
RABV-N: RABV nucleoprotein
