# Supplementary material for: Langerhans cells as targets of rabies virus immune evasion
Source: J Gen Virol. 2026 Sep 17;107(9):002318. doi: 10.1099/jgv.0.002318 (PMC13585407; doi:10.1099/jgv.0.002318)
Supplement: Supplementary file 1 [file jgv-107-02318-s001.pdf]

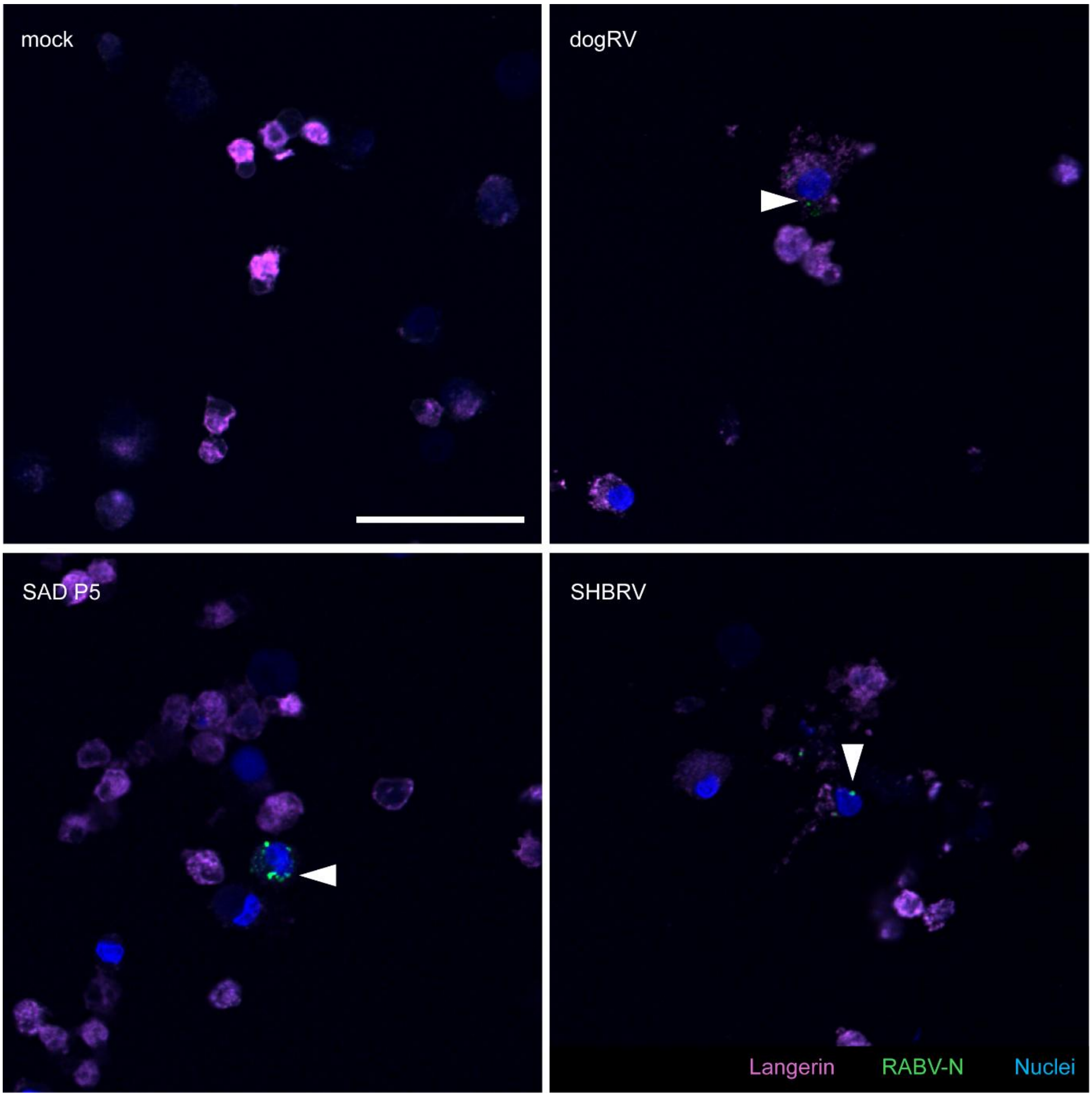

Figure S1: Internalization of RABV by LCs. Presence of RABV antigen in Langerhans cells. Cells were fixed 48 h post-infection and stained for Langerin (magenta) and RABV nucleoprotein (RABV-N, green). Virus strains dogRV, SAD P5, or SHBRV were added at an MOI of 10. Arrows indicate positive RABV-N staining. Scale bar 50 µm.

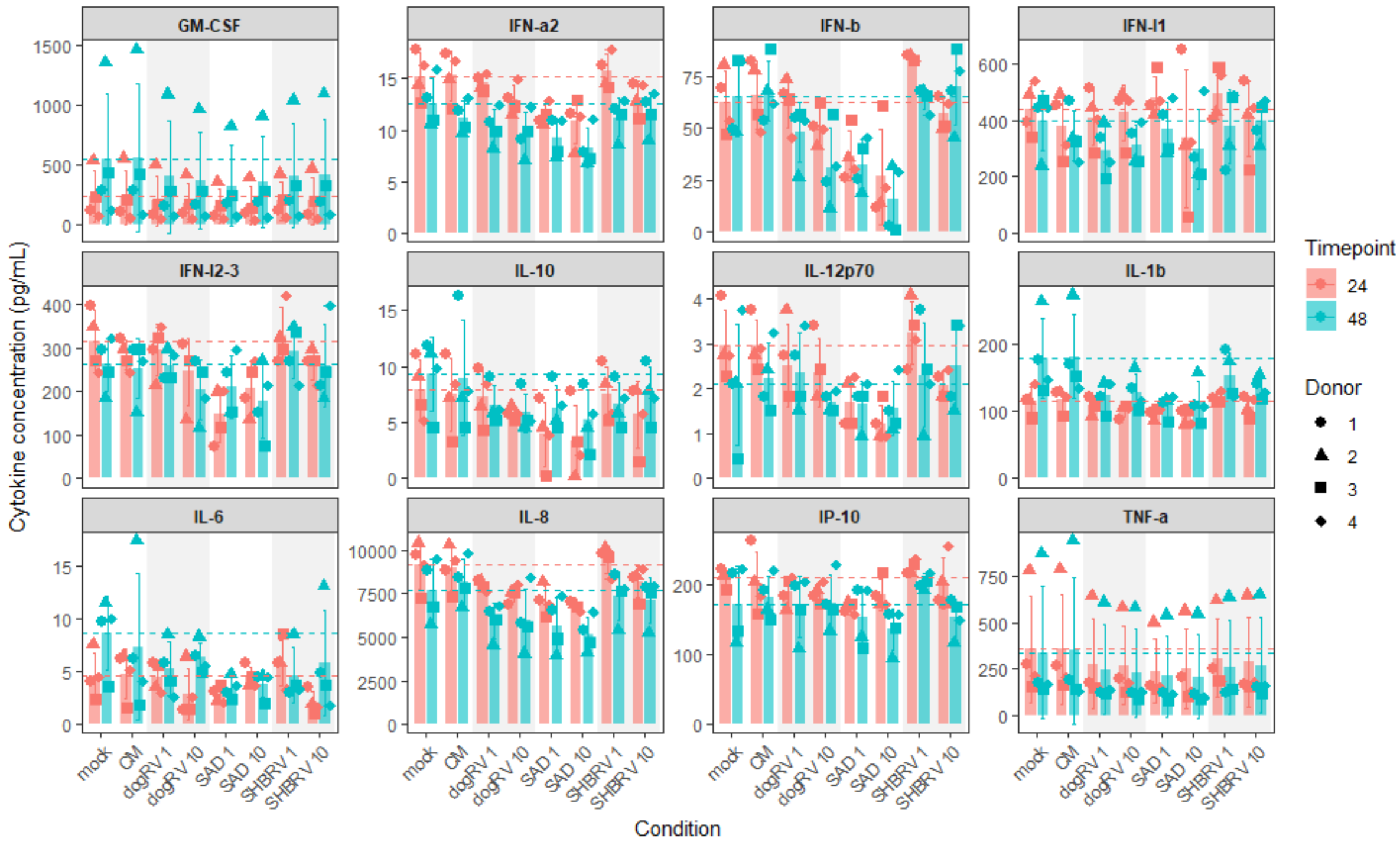

Figure S2: cytokine production by LCs in response to RABV exposure. LCs were exposed to the different RABV strains at MOI 1 and MOI 10 and supernatants were harvested at 24 h (red) and 48 h (blue) post-infection. Dashed lines represent the average cytokine concentrations in the supernatants of mock-infected LCs for each timepoint. Shapes correspond to individual donors (*n* = 4). CM, conditioned medium.
